# Maps, trends, and temperature sensitivities—phenological information from and for decreasing numbers of volunteer observers

**DOI:** 10.1007/s00484-021-02110-3

**Published:** 2021-03-10

**Authors:** Ye Yuan, Stefan Härer, Tobias Ottenheym, Gourav Misra, Alissa Lüpke, Nicole Estrella, Annette Menzel

**Affiliations:** 1 Ecoclimatology, Department of Life Science Systems, TUM School of Life Sciences, Technical University of Munich, Freising, Germany; 2 School of Biological, Earth and Environmental Sciences, University College Cork, T12K8AF, Cork, Ireland; 3 Department of Geography, University College Cork, T12K8AF, Cork, Ireland; 4 Institute for Advanced Study, Technical University of Munich, Garching, Germany

**Keywords:** Phenological season, Map interpolation, Multiple linear regression, Inverse distance weighting, Leave-one-out cross validation, Citizen science

## Abstract

Phenology serves as a major indicator of ongoing climate change. Long-term phenological observations are critically important for tracking and communicating these changes. The phenological observation network across Germany is operated by the National Meteorological Service with a major contribution from volunteering activities. However, the number of observers has strongly decreased for the last decades, possibly resulting in increasing uncertainties when extracting reliable phenological information from map interpolation. We studied uncertainties in interpolated maps from decreasing phenological records, by comparing long-term trends based on grid-based interpolated and station-wise observed time series, as well as their correlations with temperature. Interpolated maps in spring were characterized by the largest spatial variabilities across Bavaria, Germany, with respective lowest interpolated uncertainties. Long-term phenological trends for both interpolations and observations exhibited mean advances of −0.2 to −0.3 days year^−1^ for spring and summer, while late autumn and winter showed a delay of around 0.1 days year^−1^. Throughout the year, temperature sensitivities were consistently stronger for interpolated time series than observations. Such a better representation of regional phenology by interpolation was equally supported by satellite-derived phenological indices. Nevertheless, simulation of observer numbers indicated that a decline to less than 40% leads to a strong decrease in interpolation accuracy. To better understand the risk of declining phenological observations and to motivate volunteer observers, a Shiny app is proposed to visualize spatial and temporal phenological patterns across Bavaria and their links to climate change–induced temperature changes.

**Supplementary Information:**

The online version contains supplementary material available at 10.1007/s00484-021-02110-3.

## Introduction

Phenology plays an important role in ecosystem processes and functioning. It is one of the clearest and most responsive bio-indicators of climate change (IPCC 2007; Menzel et al. 2006), which is particularly vivid besides temperature measurements (Anderson et al. 2013). Phenological data especially by observers on the ground and in situ measurements have been collected worldwide, contributing to a series of long-term phenological records for climatic research studies such as trend analysis and validation for remote sensing and imaging (Izquierdo-Verdiguier et al. 2018), as well as for pollen and agrometeorology (Chuine et al. 2004; Migliavacca et al. 2012) and citizen science, since the majority of observers in these networks are volunteers (citizen scientists) (Lehmann et al. 2018). Ground observations are valuable since they start from observing responses of individual species, later on sum changing patterns or even (warming) trends from the long-term series, and finally end up with assessing consistency among dominant phenology (Badeck et al. 2004). Based on these data, many studies have shown the attributable link from climate variability to phenology, particularly referring to the occurrence dates of earlier (i.e., advanced) flowering and other spring onset phases (e.g., Chmielewski et al. 2004; Menzel et al. 2020; Rafferty et al. 2020). For instance, Rosenzweig et al. (2007) summarized in the IPCC Working Group II Fourth Assessment Report a clearly advancing trend of 0.23 to 0.52 days year^−1^ for spring phases in the 30 years prior to that study. In Europe, the spring and summer phenological events were earlier by 0.25 days year^−1^ on average from 1971 to 2000 (Menzel et al. 2006), while Menzel et al. (2020) found a slightly less advancing trend of 0.24 days year^−1^ for leaf unfolding and flowering in Germany but detected more portions of negative trends (around 90%) during 1951–2018. At the same time, prolongation of the phenological growing season has been identified not only for trees and shrubs (Fridley 2012) but also for fruit trees and crops (Chmielewski et al. 2004; Chmielewski and Rötzer 2002; Menzel et al. 2020).

Systematic phenological observations and networks have been well established over the globe with particularly numerous historical records across Europe (Schnelle 1955) such as the International Phenological Gardens (IPG) of Europe (Chmielewski et al. 2013; Menzel and Fabian 1999) and the Pan European Phenological (PEP725) database for a joint European infrastructure (Templ et al. 2018). In Germany, the National Meteorological Service (Deutscher Wetterdienst, DWD) is responsible for managing the observation network which is well equally distributed over whole Germany (Bissolli et al. 2005), maintaining the long-term phenological database (Kaspar et al. 2014), as well as presenting data visualization to the public (Kaspar et al. 2013). However, the observation numbers in the voluntary phenological network have experienced a decreasing trend since 1966 with a maximum of approximately 3700 observers (Bissolli et al. 2005; Wittich and Liedtke 2015). Similarly, the number of observers in other networks, such as in Austria, is also declining and requires concerted action. Large concerns should be addressed on such declining records, as long-term time series with adequate sample sizes are fundamental for systematic statistical analysis, as well as consistency and homogeneity in plant phenology across space and time (Brugnara et al. 2020; Bush et al. 2017). Climatic parameters, especially temperature as the main driver for phenology (e.g., Cramer et al. 2014; Fu et al. 2015), are also variable across space and time so that the interactive relationship can be better studied if desirably long and widely distributed phenological data are gathered.

In order to evaluate consequences from decreasing numbers of observations, mapping phenology via spatial interpolation could be considered, which allows visualization of phenological changes from the point to regional (continental) perspective (Gerstmann et al. 2016). Regarding individual ground observations, spatial interpolation or extrapolation must be performed when analyzing the phenological phases in space due to the importance of regional peculiarities (Dose and Menzel 2004; Jochner et al. 2013). Meanwhile, map interpolation is also able to validate and provide phenological reference for single observations, as large-scale climatic circulation patterns usually dominate the local phenological responses considering additionally geographical influences of latitude, longitude, and altitude (Bissolli et al. 2005; Ziello et al. 2009). At the same time, the map interpolation provides more insights into likely phenological trends when gaps in phenological observations hamper a solid analysis, since with few observations throughout long-term periods only the resulting fragmentary trend would not be meaningful nor representative (Bush et al. 2017). Furthermore, reliable phenological information derived from map interpolation with confidence is beneficial for models in predicting future scenarios (Migliavacca et al. 2012) and climate reconstruction (Ge et al. 2014), also as Wolkovich et al. (2012) suggested to implement interpolated observation data for building and evaluating experimental data–driven models.

The two main research questions of this study are (1) how is the performance of the phenological map interpolation impacted by varying number of observations/observers and (2) how do the long-term phenological time series spatially and temporally change when comparing observations versus interpolated products. The interpolated product was produced following a similar interpolation technique developed by the German Meteorological Service (Deutscher Wetterdiens, DWD) for climatological and phenological maps (Maier et al. 2003; Müller-Westermeier 1995; personal communication with W. Janssen). Ten phenophases representing the main phenological seasons were used based on the available phenological time series from 1951 to 2019 for the Bavarian region of Germany (Kaspar et al. 2014). Statistical analyses were performed for evaluating the map interpolation and its uncertainty using derived gridded mean onset dates and corresponding standard deviations, together with the root-mean-square errors (*RMSE*) calculated for each interpolated phenophase. The phenological trends as well as correlation analyses were calculated and compared between observations and interpolated maps. At the end, a selected observation station was compared for local agreements together with the satellite-derived normalized difference vegetation index (NDVI) and a Shiny app proposed to support volunteer observers in getting access to these types of analyses.

## Materials and methods

### Phenological observations

The phenological data are based on observations in Germany from 1951 to 2019 collected from DWD. Both annual and immediate reporters contribute to the phenological network by submitting occurrence dates throughout the whole vegetation period either once a year or immediately afterwards. Observations should be done at least twice or three times a week depending on the season. Only annual observations were considered in this study for the complete coverage of the most important species as well as clearly visible phenophases in the development cycle (Kaspar et al. 2014). Ten combinations of species and phases, i.e., phenophases, were used, representing ten phenological seasons as selected indicators for all other phenological events (see Table 1). The phenological growing season was consequently described as the period from first spring (flowering of forsythia) to winter (leaf fall of pedunculate oak) for Germany (Kaspar et al. 2014). Data for nine phenophases were available for the full time period (1951–2019) and only for the phase leaf fall of pedunculate oak (= winter) the period 1961–1990 was lacking. The detailed observation instructions and automated data quality controls had been performed by DWD, and therefore no further data filtering was made (Deutscher Wetterdienst 1991; Kaspar et al. 2014; Zimmermann and Polte-Rudolf 2013).

**Table 1:** Number of observations and uncertainty metrics for interpolated phenological seasons in Bavaria, Germany, in 2019. The number of observations (*n*) in Germany and Bavaria, respectively, are shown. The leave-one-out cross validation uncertainty (*RMSE*_*LOOCV*_) is shown as gridded averages with one standard deviation

Phenological season	Phenophase	ID	*n*		***RMSE***_***LOOCV***_ (days ± 1 × sd)
			Germany	Bavaria	
Prespring	Hazel (flowering)	113-5	969	191	9.4 ± 0.8
First spring	Forsythia (flowering)	109-5	988	192	6.3 ± 0.3
Full spring	Pedunculate oak (leaf unfolding)	132-4	897	182	7.8 ± 0.3
Early summer	Black elder (flowering)	129-5	956	188	8.0 ± 0.4
Midsummer	Large-leaved lime (flowering)	130-5	866	173	8.1 ± 0.4
Late summer	Apple early ripeness (fruiting)	311-29	652	135	12.7 ± 0.4
Early autumn	Black elder (fruiting)	129-62	887	179	10.7 ± 0.5
Full autumn	Pedunculate oak (fruiting)	132-62	613	103	11.8 ± 0.4
Late autumn	Pedunculate oak (leaf coloring)	132-31	860	168	12.6 ± 0.6
Winter	Pedunculate oak (leaf fall)	132-32	837	164	14.9 ± 0.5

### Mapping phenology

The spatial interpolation method for mapping phenology in this study is based on Hopkins’ “Bioclimatic Law”, proposing that changes in phenological onsets were dependent on latitude, longitude, and elevation (Chen 2017; Vitasse et al. 2018). In consideration of computation time and regional representation, the phenological interpolated maps in this study were produced for all ten phenological seasons in Bavaria of Germany from 1951 to 2019, following an interpolation routine of climatological and phenological maps by DWD. First of all, annual phenological observations in day of the year (DOY) were retrieved together with the geographical information of the observers’ sites. The whole area of Germany was divided into 30 overlapping equal-sized circles (five in latitude by six in longitude) with a radius of 1.95° (see Supplementary Figure S1). Multiple linear regressions were fitted for the onset date *DOY* depending on topographic elevation *h*, longitude *lon*, and latitude *lat* for each circle based on the phenological observations covered.

$$ DOY={a}_0+{a}_1\ast h+{a}_2\ast lon+{a}_3\ast lat, $$

where the corresponding regression coefficients *a*_0_/*a*_1_/*a*_2_/*a*_3_ were assigned to the centered point of each circle, and later used for inverse distance weighting (IDW) interpolation (with power of 1) for all available grid points based on their surrounding circles involved (possibly two to four circles, see Supplementary Figure S2). The interpolation was performed with the multiple linear model in terms of interpolated regression coefficients and based on the digital terrain model (Digitales Geländemodell Gitterweite 1000 m, DGM1000) with grid width of 1000 m for the topographic altitude for all grid points, and information regarding the mapping boundaries (from state to district level) was derived and mapped based on the administrative areas (Verwaltungsgebiete, VG1000), which were available on http://www.bkg.bund.de (© GeoBasis-DE/BKG 2020). Due to the lack of phenological observations for elevated regions (area size less than 1% of Bavaria), this study only focuses on the areas with elevations lower than 1000 m above sea level (a.s.l.) with 70,609 1-km grid points.

### Interpolation validation

Interpolated phenological maps were validated by the root-mean-square errors *RMSE*. To assess the multiple regression fits of circles as well as the predictive interpolation performances, the leave-one-out cross validation *LOOCV* was used for all 30 circles by calculating the circle-specific *RMSE*. The multiple regression models for each circle were trained on all available phenological observations in the corresponding circle except for the *i*th one. The test errors were averaged after all observations had been excluded once as *RMSE*_*LOOCV*_ and assigned to the centered points.

$$ {RMSE}_{LOOCV}={\left[\frac{1}{n}{\sum}_{i=1}^n{\left({P_i}^{\left(-i\right)}-{O}_i\right)}^2\right]}^{\frac{1}{2}}, $$

where *P*_*i*_^(−*i*)^ denotes the predicted *DOY* excluding the observed *DOY* at point *i*, while *O*_*i*_ denotes the observed *DOY*. The resulting *RMSE*_*LOOCV*_ were further spatially interpolated across all grid points following the same IDW routine as for multiple linear regression coefficients of phenological observations.

We also studied the potential influence of reduced observation numbers on the uncertainty of interpolated phenology in a bootstrap method. This was done by randomly selecting certain percentages of the available phenological observations (10%, 20%, …, 90%) and then calculating the respective *RMSE*_*LOOCV*_ for each interpolation circle. For each percentage level, the random selection and calculation of *RMSE*_*LOOCV*_ were repeated 1000 times to derive an averaged *RMSE*_*LOOCV*_. No manual controls of the spatial distribution (whether equally distributed or not) of the phenological observations in each interpolation circle were done in order to mimic the real observing situation.

### Statistical analyses

The interpolated phenological onset dates were visualized as anomalies which were determined as onset dates subtracted from the gridded 2019 values averaged over all available grid points (70,609) in Bavaria, for each of ten phenological seasons. The long-term phenological trends for each phenological season across Bavaria, Germany, from 1951 to 2019 were calculated and compared between in situ observations (Obs) and interpolated maps (Interpol) by using the Theil-Sen slope estimator (Sen 1968; Theil 1992; Xu et al. 2018). For interpolated maps, all available grid points (70,609) were extracted from 1951 to 2019 and treated separately as individual time series, while only observations with time length ≥ 30 years were considered (except for winter ≥ 15 years).

The Spearman’s rank correlations were calculated for onset dates of all ten phenophases in relation to respective air temperature. Grids of monthly averaged daily air temperature (2 m) over Germany from DWD Climate Data Center (CDC) were used, being aggregated into an averaged air temperature raster layer of grids (same as interpolation) from the temperature of the current, first, and second preseason months of the onset dates for each phenophase.

A selected observation station with a nearly full phenological record over 1951–2019 for the studied phenophases, Burgbernheim (Ph) (49.45° N, 10.32° E, 350 m a.s.l.), was chosen to show the local differences between the phenological observations and interpolated values regarding *R*^2^ of simple linear regression. For this site, ground-level phenological onset dates were compared with satellite indices start of the season (SOS) and end of the season (EOS) using phenological seasons first spring and winter, since high correlations were found between green-up and flowering dates (Delbart et al. 2015) as well as between threshold NDVI value and the end date of the growing season (Chen et al. 2001; Liang et al. 2011). The 16-day MODIS maximum value composite NDVI data (MOD13Q1 product) was downloaded for the years 2001–2019 using the MODISTools package in R (Tuck et al. 2014). The NDVI data has a spatial resolution of 250 m and is corrected for atmospheric and bidirectional reflectance anomalies (Didan 2015). Additionally, the corresponding pixel reliability information layer that describes the overall pixel quality (e.g., good, marginal, snow/ice, and cloudy) was downloaded using the R package. Only those observations marked as good and marginal were retained. Missing data in time series were filled using the mean annual profile values, otherwise known as climatology (Kandasamy et al. 2013; Zhang et al. 2017). The NDVI time series was then smoothed and interpolated to daily values using a LOESS function (Hufkens et al. 2019). Subsequently, the start and end of season were calculated using the widely cited half-amplitude method (Fisher et al. 2006; Misra et al. 2016; White et al. 2009).

All analyses including map interpolation and visualization were performed using *R* programming language (R Core Team 2020).

## Results

### Annual numbers of phenological observations

**Fig. 1:**
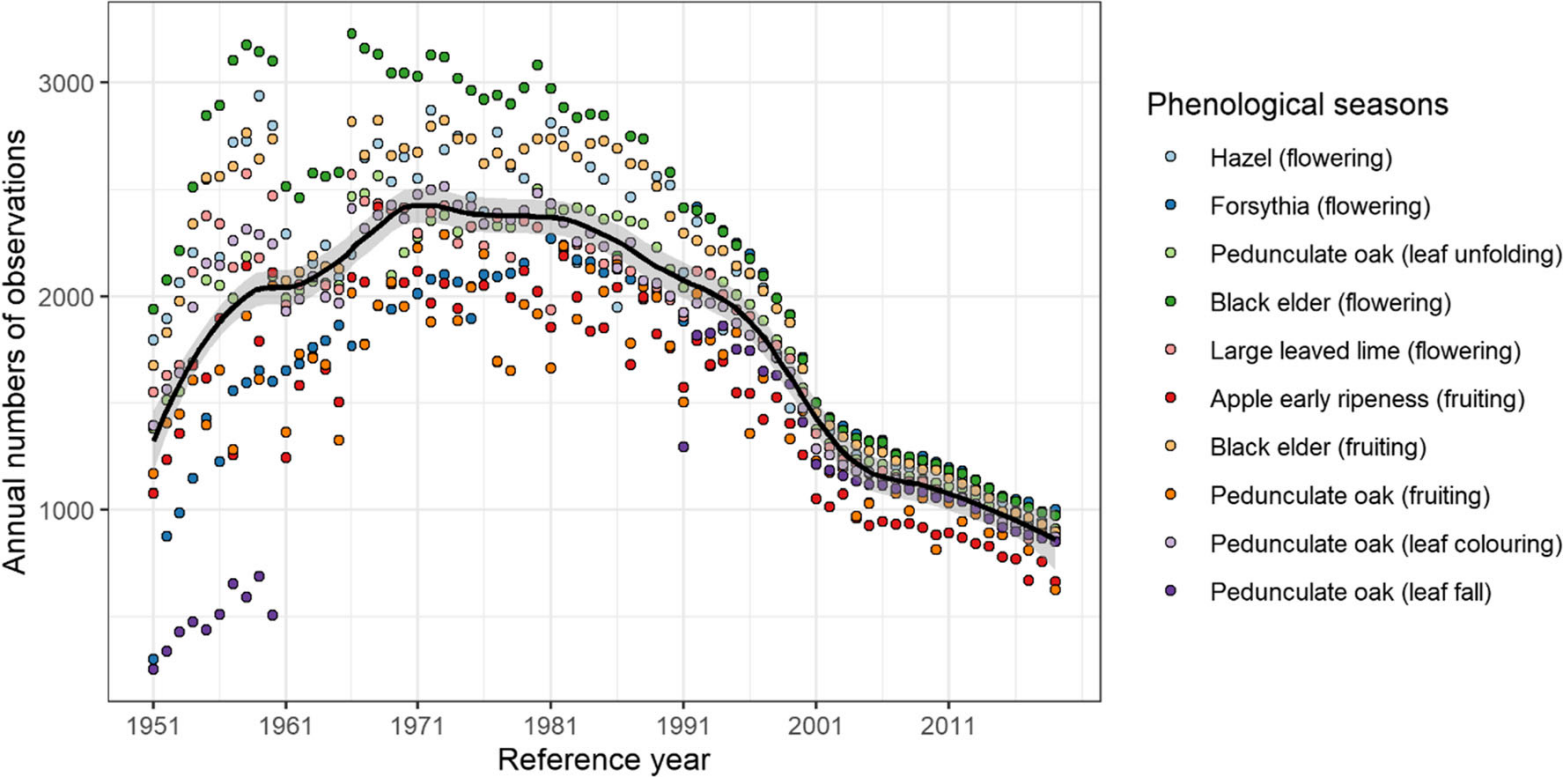
Annual numbers of phenological observations for ten phenological seasons in Germany from 1951 to 2019 collected from the German phenological observation network (DWD). Loess smoothing is applied to all numbers showing decreasing trend since 1970s

Across 69 years, there were in total 6576 phenological annual observers (both active and inactive) all over Germany, with 1171 being located in Bavaria. In 2019, for whole Germany, only 1034 observers have actively reported phenological events of the studied phenophases shown in Fig. 1. All ten phenological seasons exhibited similar changes in annual numbers of observations. The number of annual observations increased from the beginning until 1960s to 1970s (max: *n* = 3231, flowering of black elder in the reference year 1966), while it decreased afterwards, more considerably in the late 1990s. The decreasing trends were weakened since 2000 but still traceable until the end of the record. From 2001 to 2019, simple linear regressions on the annual numbers of observations resulted in similar decreasing trends for all phenophases, ranging from −28.4 observations year^−1^ (fruiting of black elder) to −19.6 observations year^−1^ (fruiting of early ripening apple varieties).

### Interpolated phenological seasons and uncertainties in Bavaria 2019

**Fig. 2:**
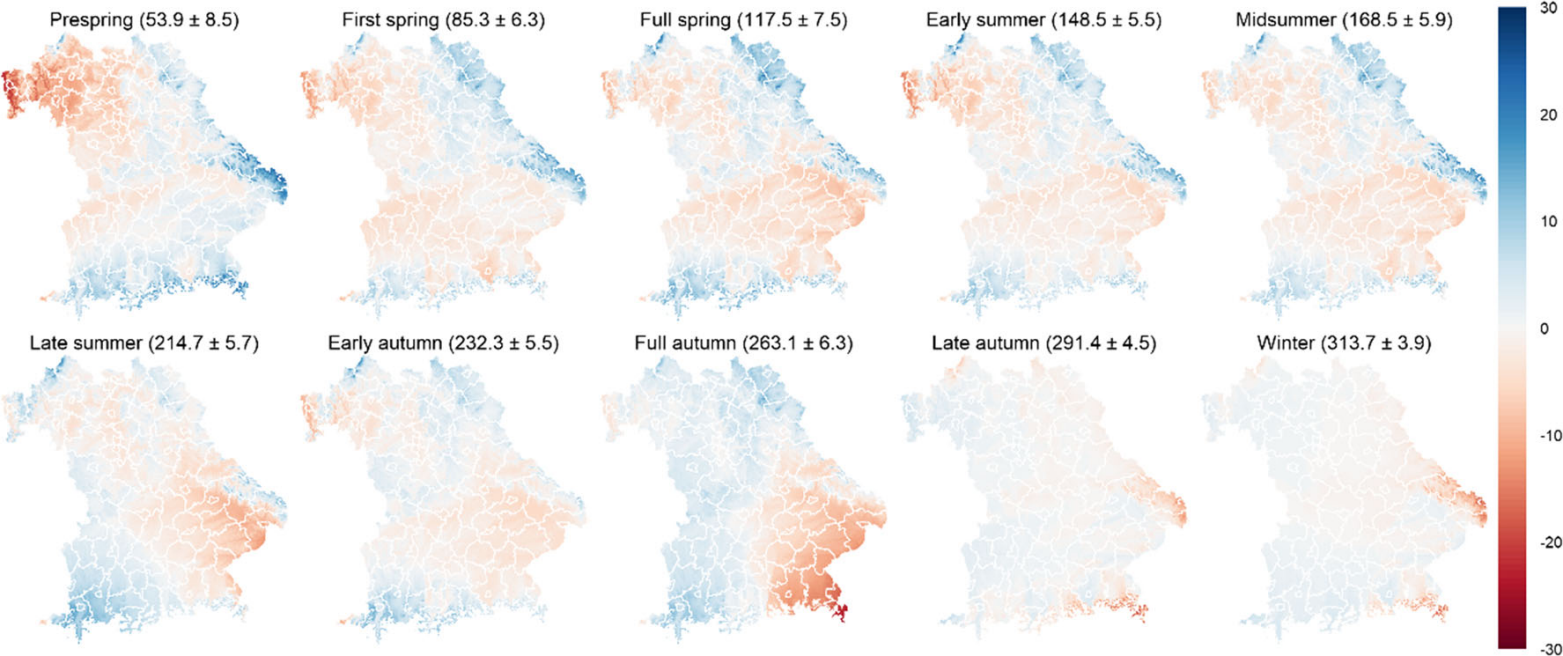
Anomalies in days as interpolated phenological onset dates subtracted from gridded means averaged over all available grid points (70,609) in Bavaria, Germany, 2019 for each of ten phenological seasons. Numbers in brackets present the gridded averaged onset dates in DOY ± one standard deviation. Negative values are shown in red for advances of onset dates and positive values in blue for delays as compared with the respective Bavarian gridded means

The spatially interpolated maps for the Bavarian region of 2019 revealed gridded mean onset dates for the ten phenological seasons ranging from DOY 53.9 to 313.7 (see Fig. 2). In prespring, the earliest spots in Bavaria are found in the warm areas of lower Franconia in the northwest. For first to full spring, the areas in lower Bavaria in the southeast speed up in their development until late summer when these areas nearby the Danube River are the first in their seasonal development. In full autumn, the southeast of Bavaria is characterized by the earliest onset dates, whereas in late autumn and winter, higher elevated areas in the Alps and the Bavarian Forest to the east experience earliest leaf coloring and leaf fall. Thus, in spring and late autumn, temperature variations with elevation may be responsible for the spatial variation between low lying areas in the northwest and highest altitudes in the Bavarian Forest and the Alps. On the other hand, the spatial variability of phenology varied with seasons, as the highest standard deviation was found in prespring (8.5 days) and the lowest in winter (3.9 days). And as a result, the phenological growing season from first spring to winter could be calculated from the interpolated maps lasting around 230 days in 2019, which was nearly 30 days longer than the growing season in 1951.

In Table 1, the lowest interpolation uncertainty was found for first spring (6.3 days) and the maximum for winter (14.9 days). Early phenophases (prespring to midsummer) always exhibited lower values than later phenophases (late summer to winter). The respective averaged *RMSE*_*LOOCV*_ for early and later phenophases showed a difference of 4.6 days, which was much larger than the spatial variability of *RMSE*_*LOOCV*_ w.r.t. one standard deviation of each phenophase (0.3–0.8 day) showing a clear seasonal difference.

### Phenological changes from 1951 to 2019

**Fig. 3:**
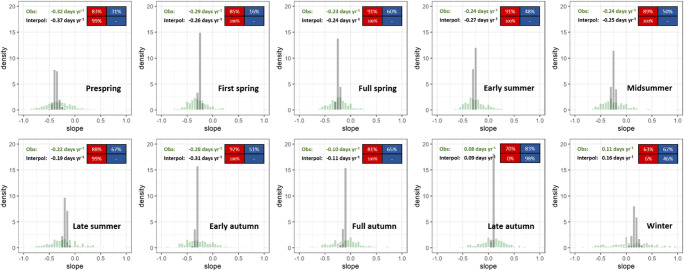
Distribution of Theil-Sen regression trend slopes (in days year^−1^) for both observations (green, Obs, time length ≥ 30 years, ≥ 15 years for winter) and interpolated gridded maps (black, Interpol), based on phenological observations in Bavaria, Germany, from 1951 to 2019 (except for winter from 1961 to 1990 due to a lack of data, see Fig. 1). Numbers in the header refer to the mean trends. Red blocks stand for proportions of statistically significant (*p* < 0.05) negative trends in all negative trends and blue for proportions of statistically significant (*p* < 0.05) positive trends in all positive trends

Figure 3 shows that both trend slopes (Obs and Interpol) were almost identical in their means, with slight differences (less than 0.05 days year^−1^) for all phenophases. The long-term trends exhibited more negative (i.e., advancing) for earlier phenophases than for later phases, as well as indicating positive changes (i.e., delayed phenological dates) for late autumn and winter only.

Considering the significance of trends, the proportions of significantly negative trend slopes were between 80 and 90% of the total negative trends, where the positive respective proportions varied from 16 to 65% for phenophases from prespring to full autumn (see Fig. 3). No clear patterns in proportions were found for late autumn and winter with the positive mean trends (around 0.1 days year^−1^ for both seasons). Interestingly, the trend slopes derived from interpolated grids did not only follow the above-described patterns but also presented a much more enhanced picture. Most of the significant proportions amounted to nearly 100% in the overall trend slopes (prespring to late autumn), and even in winter a clear difference (6%/46%) could be seen. With such clearly significant signs of trending, the interpolated maps seem to be very suitable for showing climate change in the regional (or potentially global) scale with respect to change in phenology.

### Correlation with temperature

**Fig. 4:**
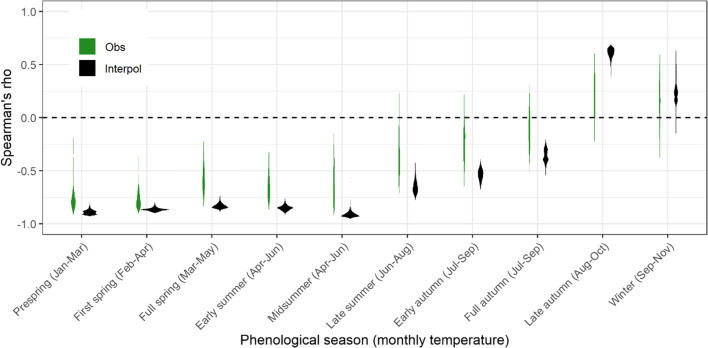
Violin plots of Spearman’s rank correlations between phenological onset dates (Interpol—interpolated maps vs. Obs—observations) for ten phenological seasons and averaged monthly air temperature (current, first, and second previous months) in Bavaria, Germany, from 1951 to 2019

From prespring to full autumn, the mean air temperature exhibited on average negative correlations with the phenological onset dates (valid for both observations and interpolated maps in Fig. 4), while in late autumn and winter, positive correlations were found (for interpolation, late autumn: 0.60 mean rank correlation; winter: 0.26). For spring and summer seasons, strong negative correlations were derived especially for interpolated phenology spanning around −0.90. And the correlations become weaker as phenological seasons progressed (for interpolation, late summer: −0.64; early autumn: −0.53; full autumn: −0.36).

### Impact of observation numbers

**Fig. 5:**
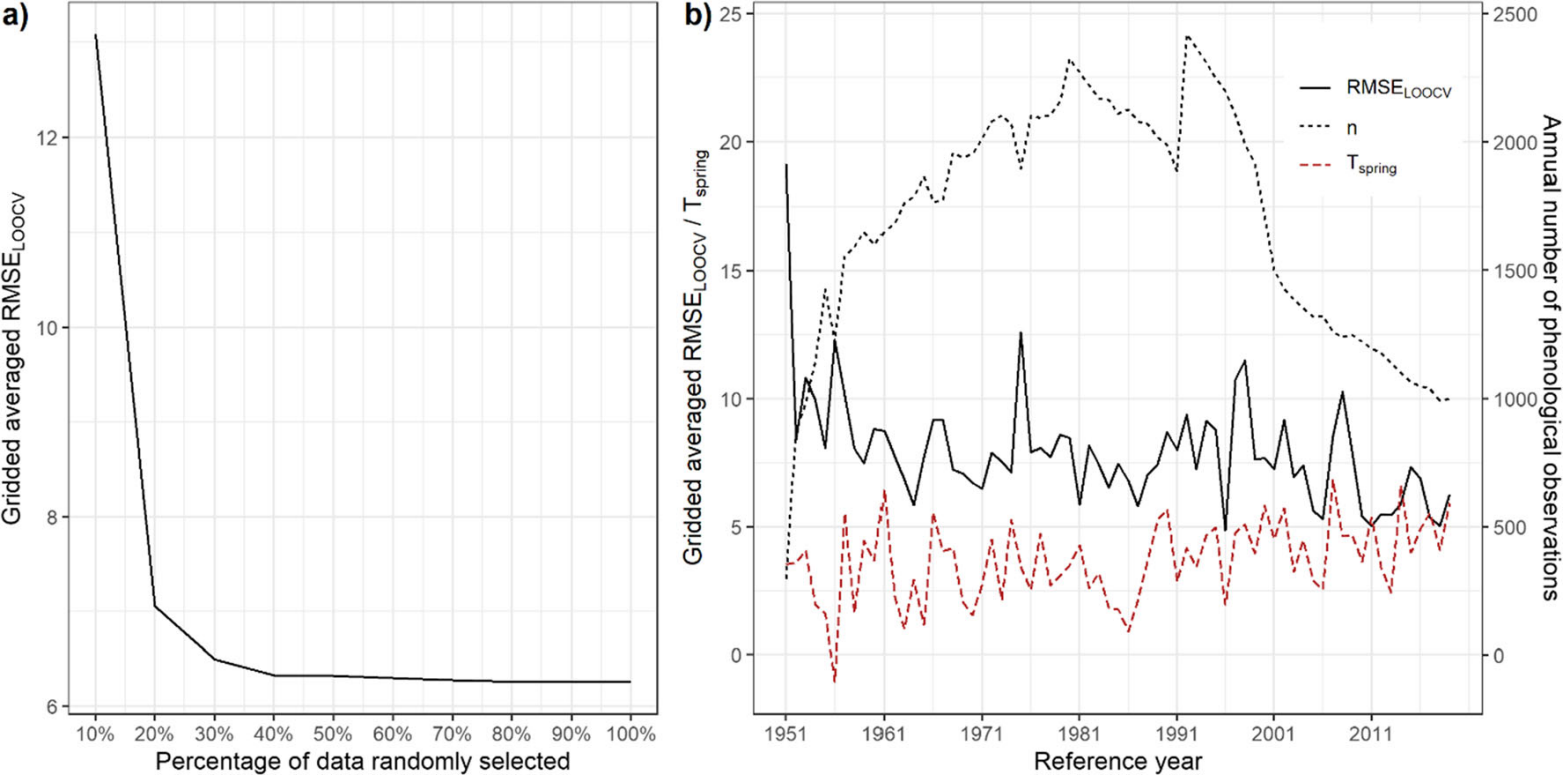
**a** Gridded averaged *RMSE*_*LOOCV*_ (in days) of increasing percentages of phenological observations randomly selected from 10 to 100% for first spring (forsythia flowering) in Bavaria, Germany, 2019. **b** Gridded averaged *RMSE*_*LOOCV*_ (in days), gridded averaged spring temperature (mean *T*_spring_ for February, March, and April, in °C), and annual number of phenological observations for first spring in Bavaria, Germany, from 1951 to 2019

Even though the numbers of observations were not related to the interpolation uncertainty for early and late phenophases (see Table 1), we were still interested in how many data (i.e., percentages of data) is needed for producing comparable good-quality interpolated phenological maps, since station density is the key for uncertainty in observation-based gridded data sets (Herrera et al. 2019). Figure 5a clearly underlines that the performance of our spatial interpolation would remain quite stable (~6.3 days) until including only 40% of the phenological observations were reached. When smaller percentages were selected, *RMSE*_*LOOCV*_ would start to increase, evidently reaching 13.1 days with 10% of selected data. Based on the available numbers of observations for first spring in 2019 in Table 1, this finding implies that there will be a higher risk of not well capturing the phenological changes when only around 300 observers were existing all over Germany (60 in Bavaria). Concerning the current decreasing trend in German phenological observing network (−26.3 observational site year^−1^, see Fig. 1), such circumstances would be met in around two decades.

Furthermore, we looked more into the temporal changes in the historical time series of first spring regarding *RMSE*_*LOOCV*_ and compared them with not only annual numbers of observations but also mean spring temperature for which gridded averaged air temperature from February to April was used. As shown in Fig. 5b, only at the beginning of the time series, the extremely high *RMSE*_*LOOCV*_ values (19.1 days) fit well with the lowest number observations available. Afterwards, the effect of observation numbers on the uncertainty seemed to be limited, since the *RMSE*_*LOOCV*_ continually exhibited a slightly decreasing trend with fluctuations. On the other hand, a continuing warming trend was observed in spring temperature, and it can also be observed that the temperature extremes could influence the *RMSE*_*LOOCV*_ to some extent, but whether it is positive/negative/lagged still remains unclear.

### Case study on point phenology

**Fig. 6:**
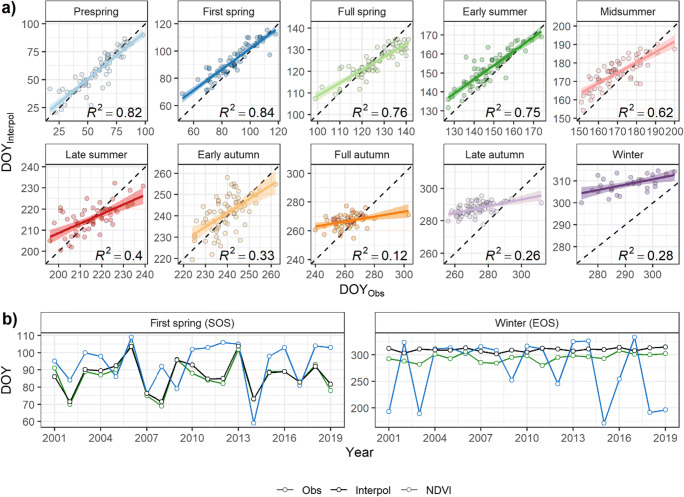
Comparison of **a** interpolated onset dates (DOY_Interpol_) with phenological observations (DOY_Obs_) for all phenological seasons from 1951 to 2019 and of **b** the normalized difference vegetation index (NDVI, SOS—first spring/EOS—winter) with ground-level phenology indices (DOY_Obs_ and DOY_Interpol_) from 2001 to 2019 at the observation site Burgbernheim (Ph) (49.45° N, 10.32° E)

Taking a closer look on how mapping phenology differs from in situ observation on the point scale, a selected observation station Burgbernheim (Ph) with best possible data coverages for most of the phenological seasons was chosen and presented in Fig. 6a, while a further comparison for the start of season and the end of season with the remote sensing technique was made. Results mirrored *RMSE*_*LOOCV*_ (see Table 1); again better agreements between observed and modeled data can be seen in earlier phenophases (*R*^2^ ≈ 0.8) than in later ones (*R*^2^ ≤ 0.4). And the interpolated phenology always exhibited less variation than the phenological observations as supported by the highly consistent phenological trends shown in Fig. 3. Interestingly from full autumn to winter, the observed phenological events appeared to be systematically earlier than the interpolation. Regarding the satellite-derived NDVI indices, in general, good agreements were reached with the ground-level phenology indices (see Fig. 6b). For the start of season (first spring for observation and interpolation), peaks and troughs in the phenological signals were almost perfectly captured by the satellite, such as in the years 2002, 2006, 2013, 2014, and 2017.

## Discussion

### Spatial variability in map interpolation

The interpolated phenological maps indicated a coherent spatial distribution of the interpolated phenological DOYs from prespring to full autumn across the Bavaria region and its elevational gradients. Comparable spatial differences were modeled by Gerstmann et al. (2016) for shooting (DOYs 100–130) and yellow ripening (DOYs 180–220) of winter wheat across Germany where these Kriging-interpolated phenological phases also showed delays of plant development in mountainous and coastal regions. Since the interpolation method applied uses regression coefficients from multiple linear regression models which most importantly account for the elevation, this spatial difference proves to be reasonable. However, late autumn and winter exhibited not only the opposite spatial patterns in DOYs but also interestingly decreased spatial variabilities (w.r.t. standard deviations) compared with early phenophases. Schnelle (1979) already mentioned that autumn phenological events were difficult for map interpolation because no consistent relationship with altitude, latitude, and longitude existed. Such patterns could be explained by the insufficient responses (or nonlinearity relationship) of plant developments to topographic conditions or parameters varying with topography in later phenophases (Hwang et al. 2011), which was also supported by the higher interpolation uncertainties (12.6/14.9 days) observed for late autumn and winter, respectively. Moreover, it seems to be irrelevant or at least cannot be fully explained by the numbers of observations across phenophases. Except for late summer (*n* = 652/135) and full autumn (*n* = 613/103), there are throughout 800–1000 phenological observations in Germany and 160–200 in Bavaria for spatial interpolation, respectively. Besides, a lack of phenological data for regions above 1000 m is also critical for the uncertainty assessment since interpolation in mountainous regions was evaluated to be associated with high uncertainty according to Gerstmann et al. (2016).

### Interpolation performance

Regarding the performance of interpolated phenology, similar uncertainty estimations were derived by other studies. Ziegler et al. (2020) used meteorological variables in a regional climate model to predict the flowering of forsythia with the *RMSE* of 8.6 days, while prediction uncertainties in terms of Kriging standard deviations for shooting and yellow ripening of winter wheat mainly ranged from 4 to 8 days (Gerstmann et al. 2016). When comparing phenological trends with other studies, a good accordance could also be found with trends especially of the leaf unfolding and flowering phases in Germany (Bissolli et al. 2005; Menzel et al. 2020) as well as within Europe (Fu et al. 2014; Jin et al. 2019; Menzel et al. 2006). However, major differences between phenological observations and interpolated products were revealed w.r.t. data distribution as well as proportions of statistically significant trends (*p* < 0.05). The trends on observational data exhibited much higher fluctuations across stations than the gridded interpolation across Bavaria. This result clearly underlines that the differences among temporal trends of single phenological observers/stations could be considerable due to two likely reasons: shorter (30+) time series instead of the full 1951–2019 series and observer sites with considerable microclimatic variation, perhaps also switch in observed individuals. In contrast, the spatial interpolation approach with multiple linear regression models was advantageous by transferring single observations into central integrations and then assigning the theoretically phenological onset date back to each grid point. Even more in situ observations were basis of this interpolation, as records from regions outside Bavaria which still fell into the modeled circles were taken into consideration. Therefore, from this perspective, the interpolated phenological maps are more representative for a broader scale (from point to region). Furthermore, a number of observations seem not to be decisive for interpolation performance yet until 10–20% observations remained, but spring temperature might play a role in the interpolation uncertainty especially from the observed inter-annual variations shown in Fig. 5b. In any case, such short-term variations in temperature (or anomalies) should be considered properly, since Herrera et al. (2019) already stated that the interpolation uncertainty would be more dependent on the number of stations if more significant internal variability exists in the mapping grids.

### Temperature dependence on phenophases

We observed a contrasting phenological response to temperature between early and later phenophases. This mirrors previous findings in the literature that spring onset is advanced by the prominent increase in preseason temperatures and autumn coloring is delayed by warm summers (Asse et al. 2018; Estrella and Menzel 2006). Similarly, Bissolli et al. (2005) reported correlation coefficients varying from −0.6 to −0.8 for all selected spring and summer phases in Germany and Slovakia, while a range of −0.3 to −0.7 was calculated for spring and summer phases in Poland by Jatczak and Walawender (2009). On the other hand, Jiang et al. (2020) detected a significantly positive correlation between leaf fall end dates and autumn preseason mean air temperatures at stations in temperate northern China from 1981 to 2012.

The resulting correlations showed the predominant influence of temperature to the early phenophases by their higher responses to mean temperatures as well as by the higher spatial variability of the phenological onset dates and lower interpolation uncertainty compared with later phenophases. This is also proved by the better adaptation of early phases to frost temperature (Scheifinger et al. 2003). The strong negative correlation coefficients together with high proportions of significant advancing phenological trends also clearly pointed out global warming as the main forcing driver for phenological changes, as supported by Rosenzweig et al. (2008) with similarly high percentages (~90%) of phenological data consistent with significant changes in warming found in Europe. Additionally, speaking of phenological observations, only the correlations for prespring and first spring showed comparable strong relationships as the pixels of the interpolated maps, while the correlations for other seasons exhibited quite scattered responses. Such similar prominent patterns for spring phases were also observed by Menzel et al. (2006). One potential reason is that certain local climate conditions—thus in situ (single) observations—might not well correspond to gridded averaged temperatures (Bissolli et al. 2005). However, given that temperature measurements cannot fully match the specific locations of phenological observations in the regional scale, only such interpolated temperature products were used and compared in this study. Further studies could focus on whether stronger correlations would occur when using station-wise temperature records and additional climatic variables such as moisture status, i.e., droughts, etc., equally mirroring the topographic effect, or whether more significant nonlinear response could be detected for phenological observations with more extreme environments as stated in Jochner et al. (2016).

### Local or regional representation

We also compared phenological seasons based on observations and interpolated products at a single station Burgbernheim (Ph), which matches the respective goodness of fit of 83% and 32% for the beginning and the end of growing season in Rötzer and Chmielewski (2001). The systematically earlier occurrences of the later phenophases in observations suggest the representation of interpolated maps is still insufficient, at least the triggering factors for phenological development are less accurately spatially interpolated than spring temperature. If more observations were available, ideally in the nearby or even the same interpolated grid, for the interpolation circle and thus better training the multiple linear regression model, the performance of spatial interpolation would be clearly improved. From the satellite perspective, the perfect match in phenological start of season derived from phenological and NDVI indices agrees well with Delbart et al. (2015) that the interannual variations in observed phenological onsets matched with green-up dates. The end of season (winter), on the other hand, shows a more similarly consistent pattern with the interpolated maps despite certain extreme values in the time series (deviations of 50–100 days in advance). These lower extremes were more likely related to phases such as cereal harvesting and intercropping, hinting towards a lower representation of regrowth signal due to coverage mismatch of spatial resolution problem. In contrast to the start of season, satellite-based end of season has been widely reported to be poorly corresponding with ground observations (Bórnez et al. 2020; Misra et al. 2018). Bolton et al. (2020) reported a higher *RMSE* between satellite- and phenocam-based end of season as compared with start of season, while Stöckli et al. (2008) reported low and sometimes even negative correlations between ground- and satellite-based end of season. The issue of matching phenology estimates of pixel averaged areas from satellite to ground observations of species-specific phenology, when compounded with the less understood autumn phenology, only increases the uncertainty in our models (Estrella and Menzel 2006; Gallinat et al. 2015; Stöckli et al. 2008). However, satellites provide data with a more consistent spatial and temporal coverage than ground-based observations that are often difficult to collect and are available with intermittent gaps. With the given knowledge, one could expect to have a more regional representation from the interpolated maps rather than the observations themselves.

### R Shiny app for citizen scientists

**Fig. 7:**
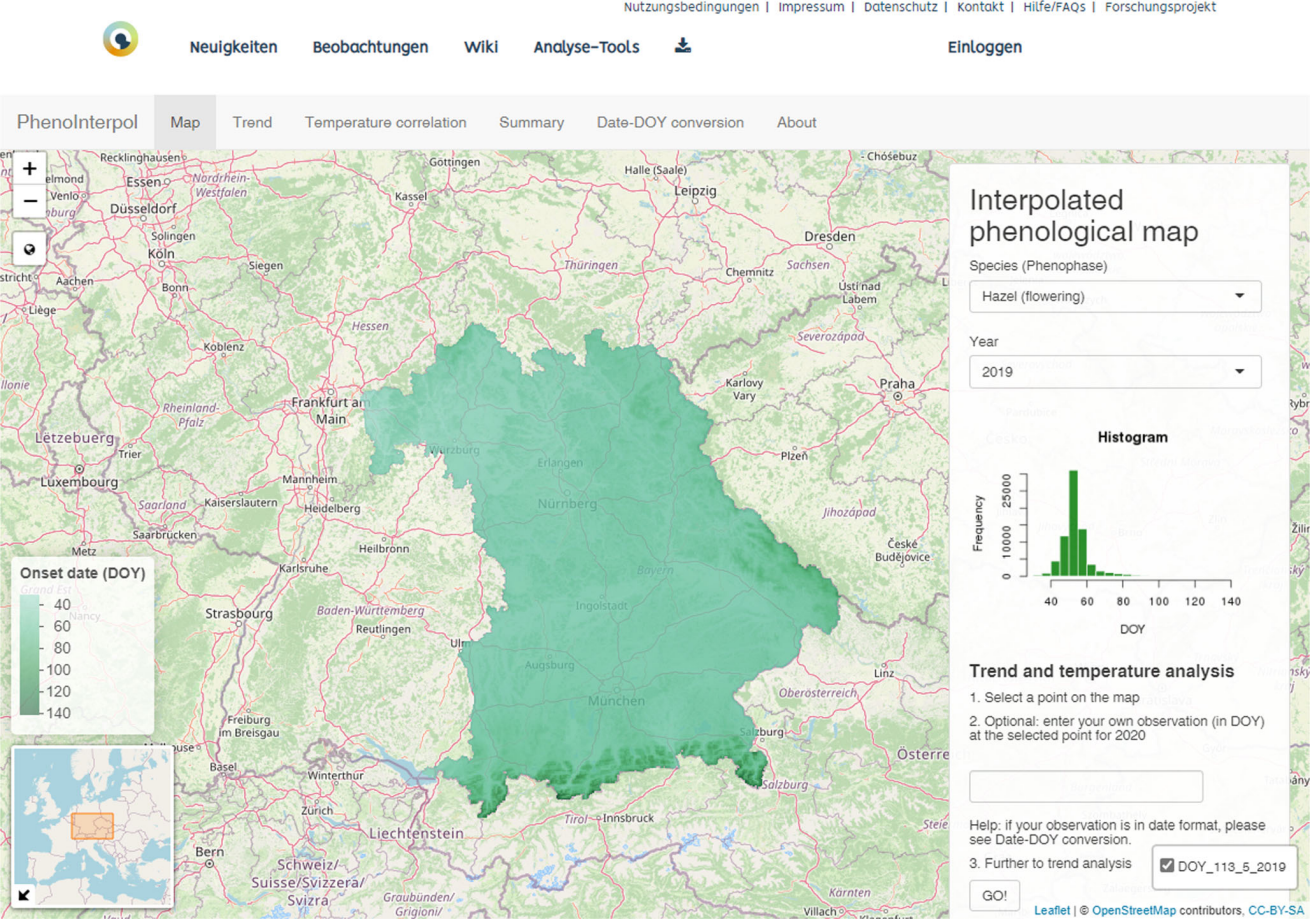
Screenshot of the R Shiny app implemented in the BAYSICS web for mapping phenology in Bavaria, Germany (available soon at https://www.portal.baysics.de/)

Visualizing the phenological changes is important and needs further contributions, especially with unforeseen climatic changing conditions as well as losing contributions from regular phenological observers. An insightful implementation of citizen scientists and decision-making communities is of great potential to gain insights attributing to climate (Delbart et al. 2015). An online interactive platform “PhenoInterpol” (see Fig. 7) was implemented for citizen science project BAYSICS—Synthesis-Information-Citizen Science Portal for Climate Change Research and Science Communication in Bavaria (Batsaikhan et al. 2020). It provides functionalities of visualizing phenological interpolated maps of desired phenophases across Bavaria (updated regularly with more species and years), collecting voluntary phenological data from users, evaluating long-term trends and preseason temperature correlations with phenological time series, and more. Most platforms only include volunteer observers in the first step of the whole process: data collection (e.g., Beaubien and Hamann 2011; Fuccillo et al. 2015). To keep observers interested and increase data quality, it is important to integrate them also in other scientific processes, e.g., asking research questions and analyzing and playing with data (e.g., Bonney et al. 2016; Kennett et al. 2015). Through tools and offers like these, volunteers grasp and appreciate the impact of their observations and feel valuable (Irwin 2018). Thus, they are not only volunteer observers but also become citizen scientists. Their voluntary work is appreciated and communicated back as important for science, making them more motivated to participate in the long run. An open communication through opportunities like research questions is started, leading to more transparency as well as an exchange of knowledge. More varieties of phenological measurements (such as phenocams) and methodologies such as breakpoint detection (Brugnara et al. 2020) and circular statistics (Rafferty et al. 2020) could further contribute to improving the mapping phenology and observation network.

## Supplementary Information

ESM 1 (DOCX 1002 kb)

## Data Availability

The phenological observations and gridded interpolated products for monthly averaged daily air temperature (2 m) over Germany are available through DWD database (https://opendata.dwd.de/).

## Code availability

The R programming code for data analyses and Shiny app can be made available by the authors, without undue reservation.
