# Supplementary material for: Maps, trends, and temperature sensitivities—phenological information from and for decreasing numbers of volunteer observers
Source: Int J Biometeorol. 2021 Mar 10;65(8):1377–90. doi: 10.1007/s00484-021-02110-3 (PMC8346396; doi:10.1007/s00484-021-02110-3)
Supplement: Supplementary file 1 — (DOCX 1002 kb) [file 484_2021_2110_MOESM1_ESM.docx]

***Supplementary material of***

**Maps, trends, and temperature sensitivities – phenological information from and for decreasing numbers of volunteer observers**

Ye Yuan^1,*^, Stefan Härer^1^, Tobias Ottenheym^1^, Gourav Misra^2,3^, Alissa Lüpke^1^, Nicole Estrella^1^, Annette Menzel^1,4^

^1^TUM School of Life Sciences (SoLS), Ecoclimatology, Technical University of Munich, Freising, Germany

^2^School of Biological, Earth and Environmental Sciences, University College Cork, T12K8AF Cork, Ireland

^3^Department of Geography, University College Cork, T12K8AF Cork, Ireland

^4^Institute for Advanced Study, Technical University of Munich, Garching, Germany

^*^**Correspondence**: Ye Yuan ([yuan@wzw.tum.de](mailto:yuan@wzw.tum.de))

**
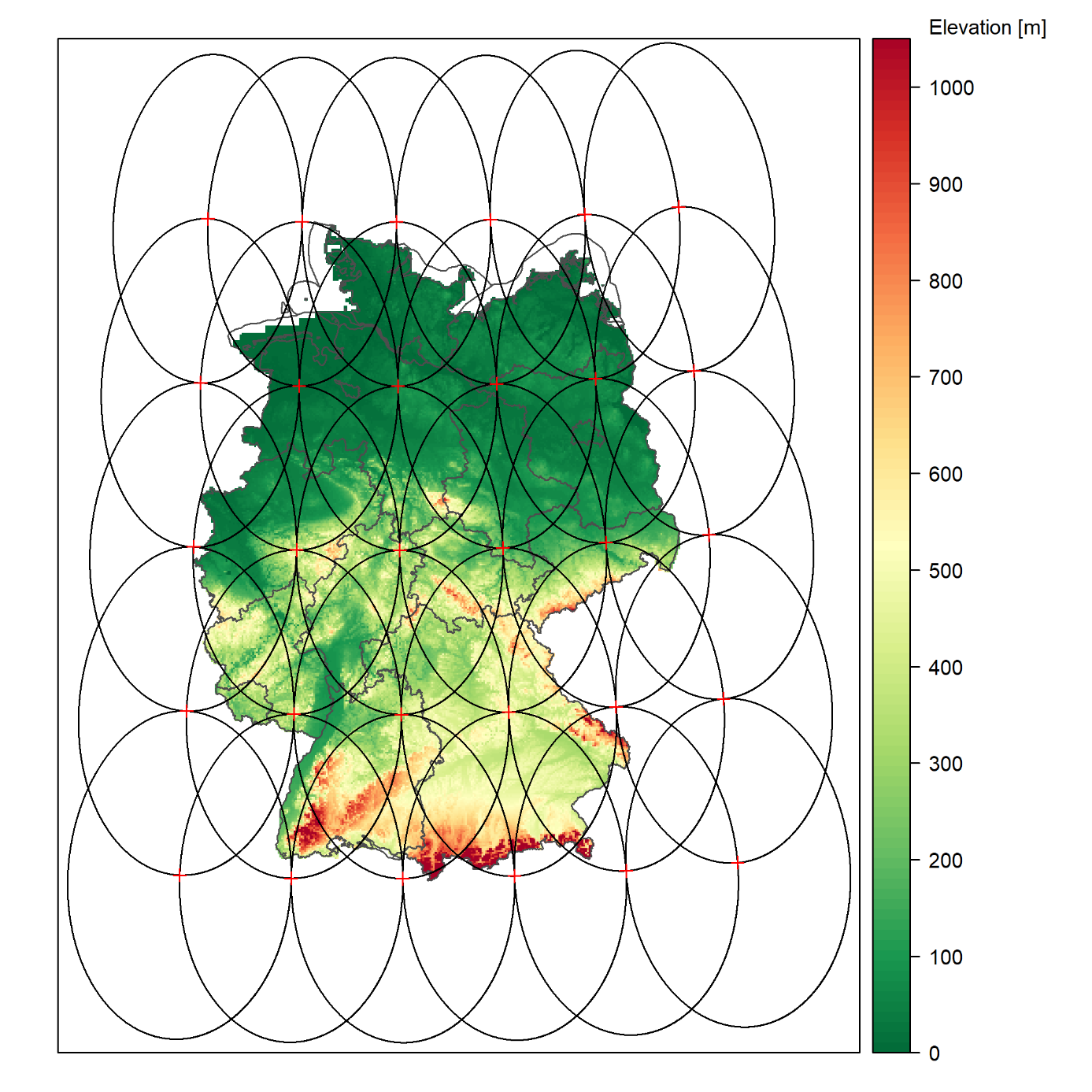
**

**Figure S1.** Illustration map of Germany for spatial interpolation covered by 30 circles (centers in red) of multiple linear regression models using phenological observations inside, based on the Digital Terrain Model (Digitales Geländemodell Gitterweite 1000 m, DGM1000), which is available on <http://www.bkg.bund.de> (© GeoBasis-DE / BKG 2020). Elevation is shown as meters above sea level.


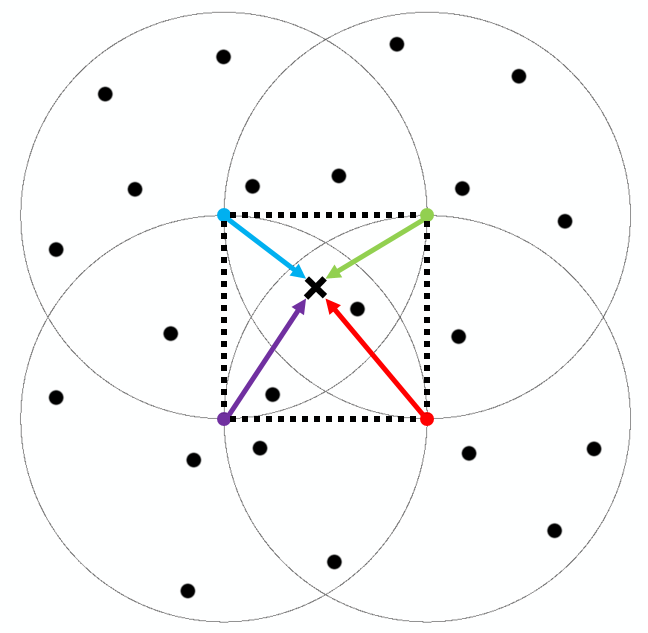


**Figure S2.** Illustration for inverse distance weighting (IDW) interpolation at selected grid points based on multiple linear regressions of phenological observations divided in circles of Germany.
